# Rolling out the radical cure for vivax malaria in Asia: a qualitative study among policy makers and stakeholders

**DOI:** 10.1186/s12936-021-03702-5

**Published:** 2021-03-23

**Authors:** Bipin Adhikari, Ghulam Rhahim Awab, Lorenz von Seidlein

**Affiliations:** 1 Mahidol Oxford Tropical Medicine Research Unit, Faculty of Tropical Medicine, Mahidol University, Bangkok, Thailand; 2 Centre for Tropical Medicine and Global Health, Nuffield Department of Medicine, University of Oxford, Oxford, UK; 3 Nangarhar Medical Faculty, Nangarhar University, Jalalabad, Afghanistan; 4 Ministry of Higher Education, Kabul, Afghanistan

**Keywords:** Radical cure regimen, Vivax malaria, Implementation, G6PD, Point of care

## Abstract

**Background:**

Wide-spread implementation of treatment regimens for the radical cure of vivax malaria is hindered by a range of factors. This has resulted in an increase in the relative proportion of vivax malaria and is an important obstacle in the achievement of global malaria elimination by 2030. The main objective of this study was to explore the current policies guiding the treatment plans on vivax malaria, and the factors affecting the implementation of radical cure in South/South East Asian and Asian Pacific countries.

**Methods:**

This was a qualitative study among respondents who represented national malaria control programmes (NMCPs) or had a role and influence in the national malaria policies. 33 respondents from 17 countries in South/South East Asia and Asia Pacific participated in interviews between October 15 and December 15, 2020. Semi-structured interviews were conducted virtually except for two face to face interviews and audio-recorded. Transcribed audio-records underwent thematic analysis using QSR NVivo.

**Results:**

Policies against vivax malaria were underprioritized, compared with the focus on falciparum malaria and, in particular, drug resistant *Plasmodium falciparum* strains. Despite the familiarity with primaquine (PQ) as the essential treatment to achieve the radical cure, the respondents contested the need for G6PD testing. Optional G6PD testing was reported to have poor adherence. The fear of adverse events led health workers to hesitate prescribing PQ. In countries where G6PD was mandatory, respondents experienced frequent stockouts of G6PD rapid diagnostic kits in peripheral health facilities, which was compounded by a short shelf life of these tests. These challenges were echoed across participating countries to various degrees. Most respondents agreed that a shorter treatment regimen, such as single dose tafenoquine could resolve these problems but mandatory G6PD testing will be needed. The recommendation of shorter regimens including tafenoquine or high dose PQ requires operational evidence demonstrating the robust performance of point of care G6PD tests (biosensors).

**Conclusion:**

There was sparse implementation and low adherence to the radical cure in South/South East Asian and Asian pacific countries. Shorter treatment regimens with appropriate point of care quantitative G6PD tests may resolve the current challenges. Operational evidence on point of care quantitative G6PD tests that includes the feasibility of integrating such tests into the radical cure regimen are critical to ensure its implementation.

**Supplementary Information:**

The online version contains supplementary material available at 10.1186/s12936-021-03702-5.

## Background

Over the last decades, a significant decline in malaria has been achieved globally and in South East Asia specifically [1]. An important reason for the increased focus on malaria elimination has been the emergence and spread of artemisinin resistant *Plasmodium falciparum* strains in the Greater Mekong Sub-region, which could potentially spread globally leading to a public health catastrophe [2]. A multi-pronged approach was intensified in the region to contain the spread of artemisinin-resistant falciparum malaria, including the increased distribution of LLINs and the early diagnosis and appropriate treatment of malaria. Funding has also been made available for novel tools such as mass drug administration, increased community engagement and strategies to target remote and at-risk populations such as pregnant women, children, forest goers and migrant populations [3–9]. These intensive efforts have resulted in a significant decline in falciparum malaria, but with a lower impact on *Plasmodium vivax* infections [10–14]. Eliminating vivax malaria is complicated because of latent liver hypnozoites that can relapse weeks to months after the initial infection [15, 16]. A schizontocidal treatment regimen used to treat falciparum/vivax malaria does not clear hypnozoites and thus the addition of an 8-aminoquinoline, such as primaquine (PQ) or tafenoquine (TQ), is required. The treatment of both schizonts and hypnozoites to prevent relapse is referred to as the radical cure [17].

The administration of 8-aminoquinolines is complicated by two factors. First, there may be poor adherence to the 14-day primaquine regimen [18]. 8-amoniquinoline regimens such as a short high dose PQ course or a single dose tafenoquine could mitigate the risk of non-compliance to a 14-day course [19]. Second, 8-amoniquinolines can cause haemolysis in patients with Glucose 6 Phosphate Dehydrogenase deficiencies (G6PDd) [17]. An efficient point-of-care quantitative G6PD test (biosensor) may allow accurate screening of G6PD activity before initiating the radical cure treatment [20]. Novel biosensor G6PD tests and newer radical cure regimens are currently under evaluation in several countries in South East Asia.

Historically, *Plasmodium vivax* has been under-prioritized by national malaria control programmes and the World Health Organization (WHO), as evidenced by the delayed inclusion of separate vivax malaria estimates in the World Malaria Report until 2013 [15]. The predominance of falciparum malaria in much of sub-Saharan Africa where most of the malaria related mortality occurs and the erroneous perception regarding the benign nature of vivax malaria have contributed to the lack of attention to vivax malaria by the global community [21]. In addition, artemisinin-based combination therapy (ACT), the first-line schizontocidal treatment recommended for falciparum malaria, also cures febrile illness due to vivax malaria, resulting in a lack of appreciation for the importance of the radical cure. Also, a detailed understanding of vivax relapse and the associated direct and indirect costs may be beyond the (immediate) purview of policymakers and clinicians. For instance, one in five vivax infections in Nepal is caused by long latency strains that could relapse after 9 months and thus requires prolonged follow-up [22]. Relapsing infections were estimated to constitute more than 80% of clinical episodes due to *P. vivax* malaria in the Asia Pacific region [23].

To date, few studies have asked policy and key decision-makers about the perceived barriers in the implementation of the radical cure therapy in the region [24]. The main objective of this study was to explore the current policies guiding the treatment plans on vivax malaria, and the factors affecting the implementation of radical cure in the region among policy makers and relevant stakeholders who have influence in the national malaria control programs in South Asia, South East Asia and Asia Pacific.

## Methods

### Design

This was a qualitative study among policymakers who either represented national malaria control programmes of their countries or were major stakeholders and had role and influence in national malaria policies such as relevant persons from non-governmental organizations. The study followed standard criteria for reporting of qualitative studies (COREQ) guidelines (Additional file 1: Appendix S1) [25]. A phenomenological approach was used to explore the topic in-depth based on the interviews.

### Study participants

A list of potential participants consisting of policymakers and relevant stakeholders was built from the network of the researchers (BA,  GRA, LvS), their institution (Mahidol-Oxford Tropical Medicine Research Unit) and professional contacts, specifically the Asia Pacific Malaria Elimination Network (APMEN). The list of participants kept growing due to recommendations from potential participants and ultimately consisted of 77 potential respondents in 18 countries. Participants were included based on their relevance to national malaria control programmes and roles and influence in the malaria policies in the region (purposive selection). All participants in the list were initially contacted by email. The sampling approach relied on the basic tenet of qualitative research, where respondents were selected/approached based on their involvement in the activity of interest, in this case their roles in malaria control programmes in the region [26]. The participants consisted of relevant stakeholders from 17 countries that included: Afghanistan, Bangladesh, Bhutan, Cambodia, India, Indonesia, Laos, Malaysia, Myanmar, Nepal, Pakistan, Philippines, Papua New Guinea, Salomon Islands, Thailand, Vietnam, and Vanuatu. A total of 33 respondents participated in this study (Table 1). Three of the potential respondents were retired and either forwarded the email or provided the email addresses of the incumbent contemporaries. One refused to participate for no stated reason but referred to a published policy for his/her perspectives. Five potential respondents could not be interviewed although they agreed to participate initially. A minimum of one key respondent from a country participated in the study. Number of respondents (sample size) for this study was based on the principles of 'data saturation', that is data were collected until no new data/themes emerged from further interviews [27].

**Table 1:** Demographic profiles of participants in this study (n = 33)

SSI#	Age range in years	Sex	Country	Qualification	Type of job
SSI-1	50–60	M	Nepal	PhD	Government
SSI-2A	40–50	M	Bhutan	MPH	Government
SSI-2B	50–60	M	Bhutan	PG Diploma	Government
SSI-3	40–50	M	Nepal	MD, MPH	Non-Government
SSI-4	40–50	M	Pakistan	MBBS, MPH	Government
SSI-5	50–60	M	Nepal	MD, MSc	Non-Government
SSI-6	40–50	M	India	MD	Government
SSI-7	60–70	M	Bangladesh	MD, PhD	Government
SSI-8	50–60	M	Bhutan	PhD	Non-Government
SSI-9	50–60	M	Cambodia	MD, MPH	Non-Government
SSI-10	50–60	M	Laos	MD, PhD	Government
SSI-11	50–60	M	Afghanistan	MPH	Government
SSI-12	50–60	M	Cambodia	MD, MPH	Non-Government
SSI-13	50–60	M	Cambodia	MD	Non-Government
SSI-14	40–50	F	Afghanistan	MD, MPH	Government
SSI-15	50–60	M	Salomon Island	MPH	Government
SSI-16	40–50	M	Pakistan	MSc	Government
SSI-17	50–60	M	Philippines	MD, MPH	Government
SSI-18	40–50	F	Thailand	MD, MCTM	Government
SSI-19	60–70	M	Indonesia	MD, PhD	Non-Government
SSI-20	60–70	M	India	MBBS, MD	Government
SSI-21	30–40	M	Vietnam	MD, PhD	Non-Government
SSI-22	60–70	F	Indonesia	MD	Government
SSI-23	60–70	M	Myanmar	MD, PhD	Non-Government
SSI-24	60–70	M	Philippines	MD, DTM&H	Non-Government
SSI-25	40–50	F	Vietnam	PhD	Non-Government
SSI-26A	50–60	M	Malaysia	MD	Government
SSI-26B	50–60	F	Malaysia	MPH	Government
SSI-27	40–50	M	Myanmar	MD, PhD, MBA	Non-Government
SSI-28	40–50	F	Vanuatu	PhD	Non-Government
SSI-29	50–60	M	PNG	MD, MPH	Non-Government
SSI-30	40–50	M	Afghanistan	MD, MPH	Non-Government
SSI-31	40–50	M	Afghanistan	MD, MPH	Non-Government

*SSI* semi-structured interview, *M* male, *F* female

### Data collection and interview guide

A semi-structured interview guide was constructed based on the overarching research questions to make the radical cure universally available in vivax endemic countries:

1. What is the most appropriate 8-aminoquinoline regimen in a given setting?
2. What is the most appropriate G6PD test?
3. What other measures are available to ensure safety?
4. Where and by whom should the radical cure be administered?

Finding solutions for the challenges implied in each of these questions is likely to hold the key to vivax elimination. All themes and corresponding research questions were refined based on the past literature [18, 24, 28, 29]. Questions and theme-guide were further revised based on the discussion among authors, followed by a discussion with relevant researchers, and stakeholders who were aware of the policy landscape in the region (Additional file 2: Appendix S2). Two interviews were piloted among researchers which informed and refined the theme (study) guide; and are excluded from the study. A total of 29 remote (virtual) interviews were conducted by BA. AGR conducted two face-to-face interviews in Afghanistan. Potential respondents were initially approached with requests for appointments by email and phone in the case of face-to-face meetings. The majority of the respondents were not acquainted with the researchers and did not have a personal relationship. All potential participants were sent a standard participant information sheet and interview guide before deciding whether to participate in the study. Due to ongoing COVID-19 pandemic, most of the interviews were conducted remotely using communication platforms (Microsoft Teams, Cisco Webex and Microsoft Skype). The interviews were only audio-recorded following the respondents’ consent. Only one respondent opted out of the audio-recording of the interview but agreed to participate in the study. Two interviews were conducted face to face, because the participants chose that option. The average duration of an interview was 35 min ranging from 25 to 60 min. All interviews were conducted in English and were transcribed verbatim for analysis. None of the transcripts were returned to participants for their comments or suggestions. The interviews were conducted between 15th October, 2020 and 15th December 2020. No repeat interviews were carried out with the participants but a few of them were consulted for clarifications.

### Data analysis

**Fig. 1:**
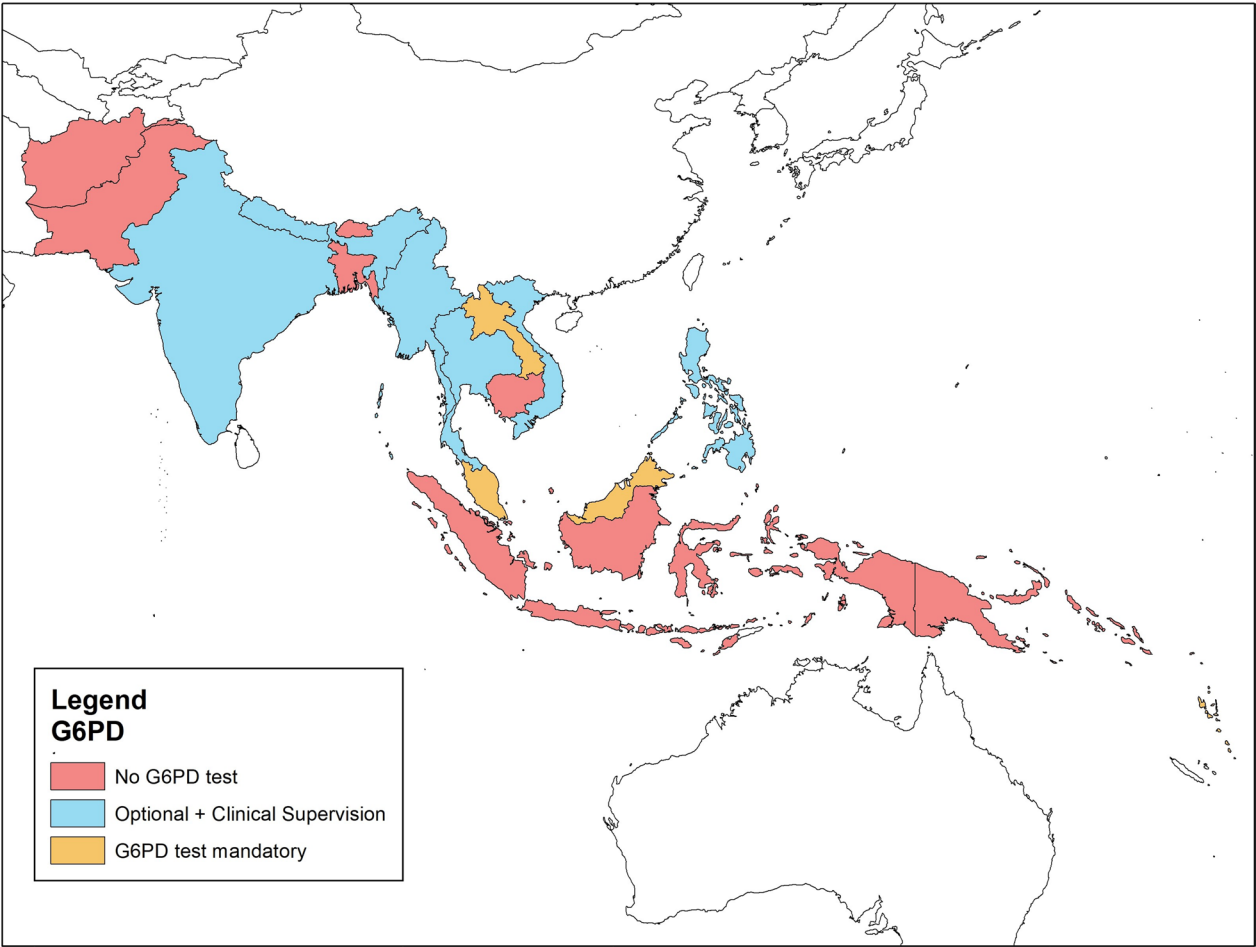
Policies on G6PD test in countries in South/South East Asia and Asia Pacific

**Fig. 2:**
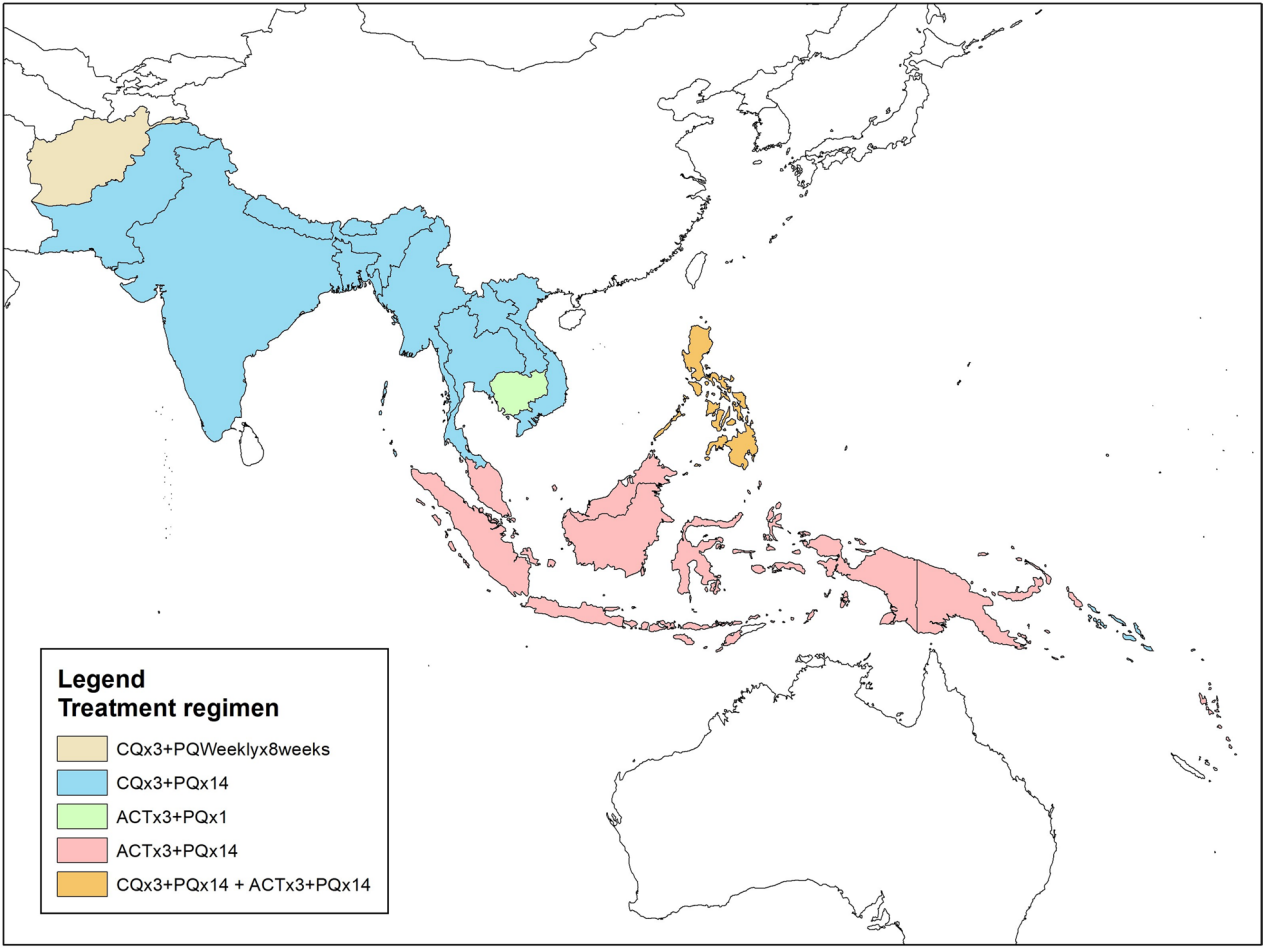
8-aminoquinolones in (radical cure) treatment regimen in countries in South/South East Asia and Asia Pacific

**Fig. 3:**
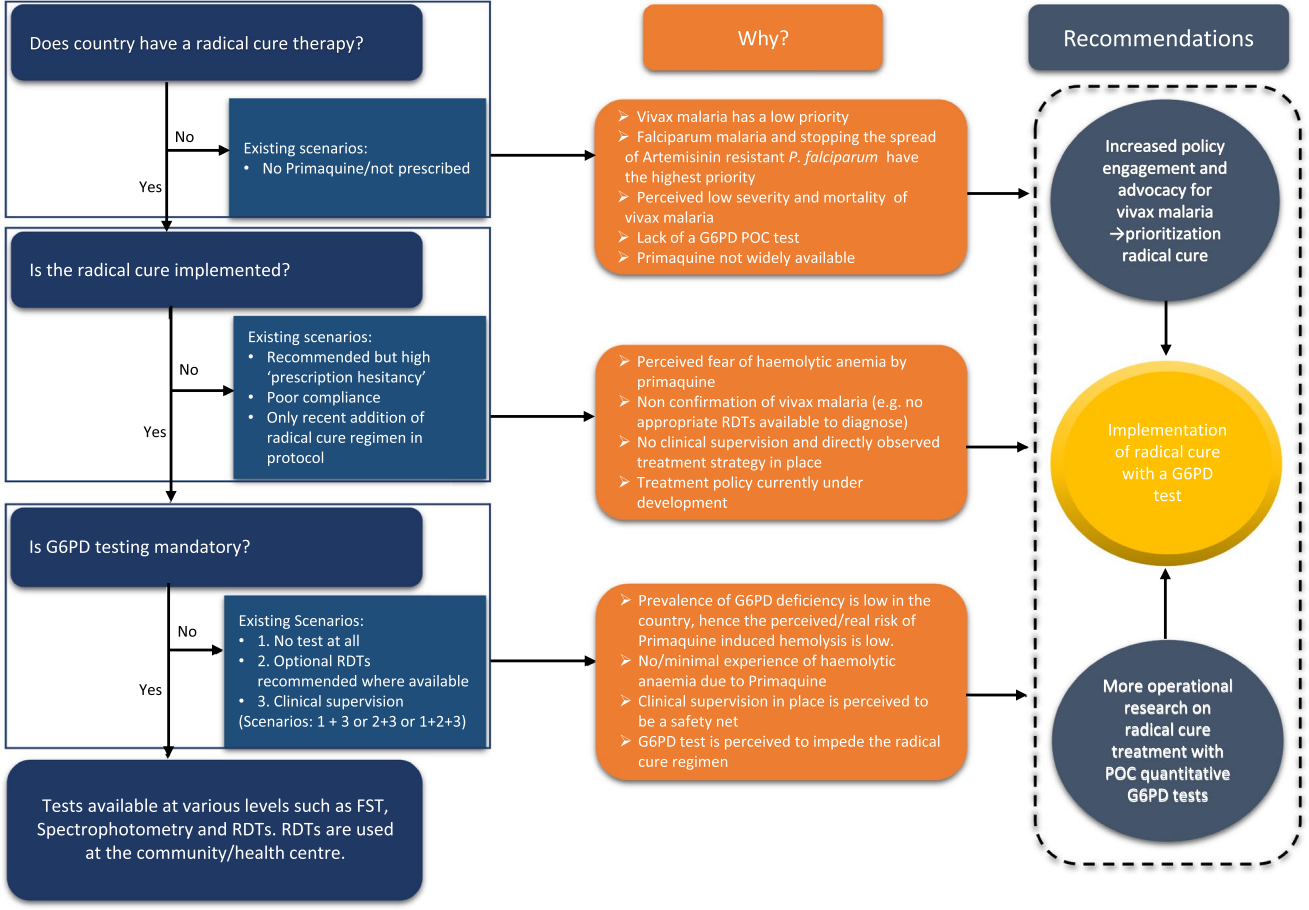
Operational challenges and barriers to roll out current radical cure regimen in South/South East Asia and Asia Pacific

**Fig. 4:**
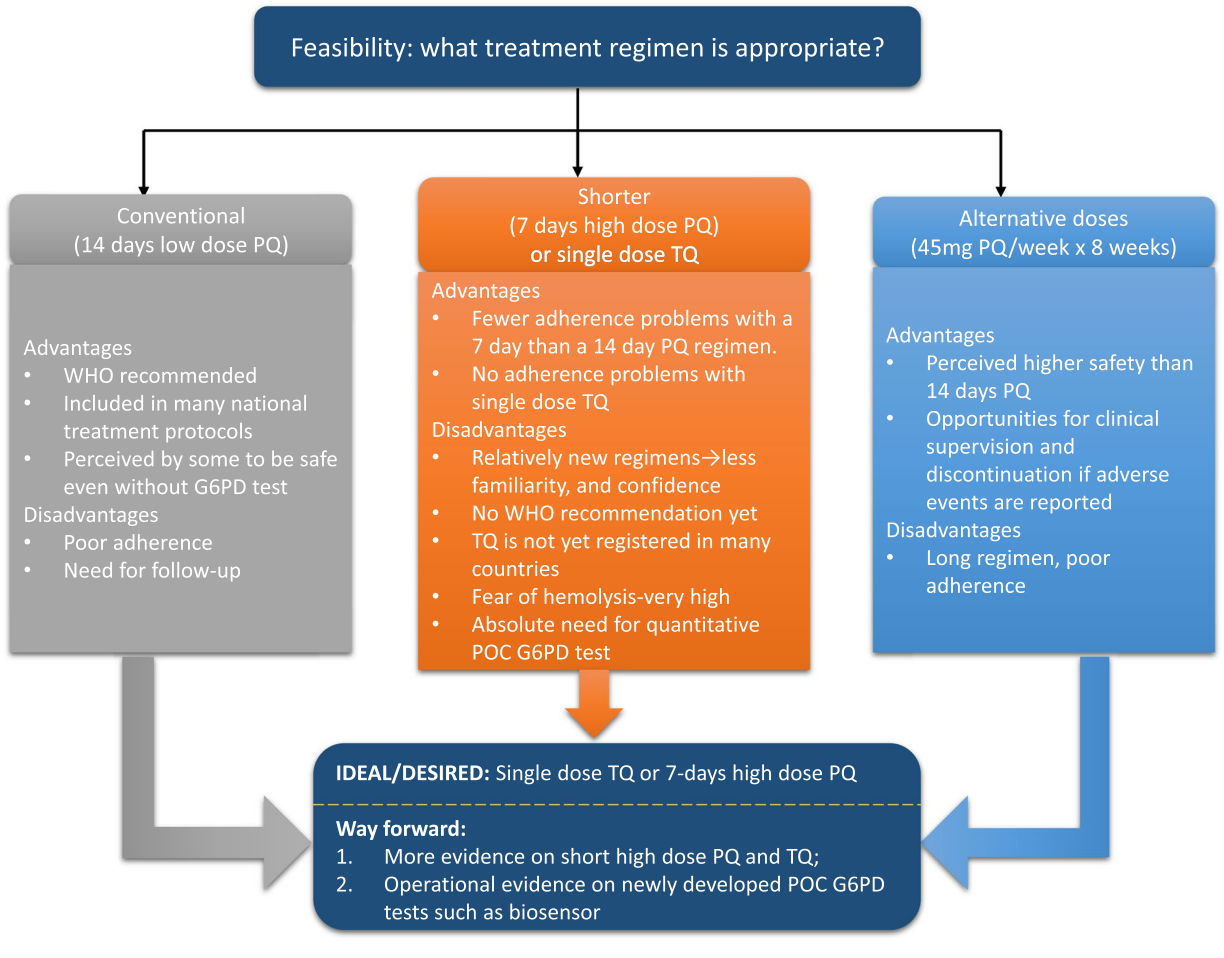
Feasibility and evidence gaps of current and future radical cure regimens of vivax malaria in South/South East Asia and Asia Pacific

All transcripts and the interviewer’s notes were first cross-checked with the audio-recordings in Excel (Microsoft, Seattle, WA, USA). Policies related to treatment regimens and G6PD tests were extracted into an Excel sheet and informed the maps and policy context for the study (Figs. 1, 2). Data from the study were explored to construct the policy landscape that summarized the heterogeneity in policy scenarios guiding radical cure regimen in various countries including reasons and future recommendations (Fig. 3). Feasibility of various radical cure regimen and evidence gaps were explored and summarized in Fig. 4. Transcripts and the notes were coded line by line in qualitative data analysis software NVivo 12 (QSR International, Doncaster, Australia). The initial codebook based on the interview guide (deductive approach) was revised according to the emerging themes from the data (inductive approach). Two investigators (BA and LvS) independently checked the coded data against the transcripts. Regular debriefings were held between the researchers concerning the final themes and their interpretation to reach a consensus. None of the participants were consulted for feedback on the findings. Themes and the supporting quotes were built based on the research question of this study and are presented below.

## Results

### Study context

Policymakers and stakeholders from 17 countries were interviewed starting with questions regarding the two fundamental aspects of vivax malaria treatment regimen, G6PD testing for routine treatment of vivax malaria (Fig. 1) and the radical cure regimens use in the country of the respondent (Fig. 2). In majority of the countries in the region (Afghanistan, Bangladesh, Bhutan, Cambodia, Indonesia, Pakistan, Solomon Islands and Papua New Guinea), G6PD tests were either not available or not mandatory for routine treatment of vivax malaria. In six countries (India, Myanmar, Nepal, Philippines, Thailand, and Vietnam), G6PD testing was not mandatory and PQ was provided with clinical supervision and follow-up. Clinical supervision ranged from directly observed treatment in Bhutan to simple counselling to discontinue PQ in case of adverse events in Nepal. In Myanmar G6PD tests are not recommended for routine care, while in India, Nepal, Philippines, Thailand, and Vietnam, G6PD tests are recommended and encouraged where available. In Laos, Malaysia, and Vanuatu, G6PD tests are mandatory. In Malaysia and in the Philippines new-borns are screened for G6PD test. Although Laos and Vanuatu made G6PD test mandatory, both countries report limited availability of test kits at the community level.

National malaria treatment guidelines recommend a range of PQ regimens, for example Afghanistan recommends combination of CQ × 3 days + PQ 0.25 mg/kg daily for 14 days but due to absence of G6PD tests uses 0.75 mg/kg weekly PQ for 8 weeks, the equivalent of 45 mg/week in a 60 kg individual. The majority of the countries (Bangladesh, Bhutan, India, Laos, Myanmar, Nepal, Pakistan, Solomon Islands, Thailand, and Vietnam) use CQ × 3 days + standard low dose (0.25 mg/kg) PQ × 14 days. Cambodia does not use a radical cure regimen. Indonesia, Malaysia, Vanuatu, and Papua New Guinea use an ACT × 3 days + (0.25 mg/kg) PQ × 14 days. Philippines uses ACT or CQ + (0.25 mg/kg) PQ × 14 days interchangeably.

### Overview

Three overarching themes were identified based on the research question and the findings in this study are categorized into: 1. Policy priority towards vivax malaria; 2. Operational challenges and barriers to roll out current radical cure regimens (Fig. 3); and 3. Evidence gaps for future radical cure regimens (Fig. 4). The respondents had a clear understanding of policies and treatment guidelines for falciparum malaria due to the associated threat of artemisinin resistance, morbidity and mortality. In contrast, vivax malaria related policy and management guidelines were perceived as more complex and were given a lower priority. This may have affected the standardization and uniform implementation of radical cure regimens. Although the respondents were familiar with PQ as a drug for the radical cure, its dosing and criteria for its implementation were contested with a sharp division between those who thought G6PD testing was mandatory before initiating radical cure, and others who thought mandatory G6PD testing is of little value and impedes the benefits by PQ. There was an apprehension and prescription hesitancy among health workers in prescribing PQ in the absence of G6PD testing which ultimately leaves radical cure regimens unimplemented. The 14-day PQ regimen (0.25 mg/kg) was the most frequently adopted national policy, however, poor adherence was realized to be a major challenge. These challenges were echoed across countries in various degrees. Most respondents agreed that a shorter treatment regimen and point of care quantitative G6PD tests could resolve the problem but asked for operational evidence.

### Policy priority towards vivax malaria

Most of the respondents in this study agreed that vivax malaria was not a priority until recently when the decline in falciparum malaria unmasked the contribution of *P. vivax* to the overall malaria burden. Vivax malaria was perceived by the majority of the respondents to be less severe and associated with less mortality compared to *P. falciparum*. For instance, in Bhutan, cases of *P. falciparum* used to be treated and monitored for parasite clearance at in-patient wards, while patients suffering from vivax malaria were sent home and not even asked to return for follow-up appointments. Several respondents explained that the concept of expediting malaria elimination emerged due to the potential spread of artemisinin resistant *P. falciparum* strains. The focus on *P. falciparum* helps explain why there was no national specific *P. vivax* malaria elimination policy. Other selected respondents contested perceptions that vivax malaria is less dangerous, and less severe than falciparum malaria.

*‘Well, there are couple of things going on here. One thing is the wrong health belief that P. vivax cannot hurt. This is the infection that cannot hurt. We now know that’s not true. But that evidence has been slow to emerge and very slow for people to accept the reality that untreated P. vivax is actually very dangerous. But the thinking that has been for decades, this is a harmless infection. The drug that we use to prevent recurrence is potentially dangerous. So, the practice in Indonesia overwhelmingly has been simply not to prescribe the treatment.’*

***SSI-19, Indonesia.***

The lack of strategies targeting specifically vivax malaria was thought to be due to the lower death rates attributable to *P. vivax* malaria than to *P. falciparum* malaria coupled with steady decline in malaria burden over the years. Few respondents explained that there was an overall decrease in the priority to control malaria ultimately affecting vivax malaria programmes including the lack of support and backup plans to study vivax malaria. Most respondents echoed how the epidemiological burden of *P. falciparum* had the highest priority in the past.  Only recently when the relative proportion of *P. vivax* began rising did *P. vivax* control programmes gain prominence. This included the adoption of 8-aminoquinolones in national treatment policies.

*Ah ya before, the most dominant species was Pf as we move[d] on, the proportion of falciparum steadily gone [went] down. But then before, we didn’t treat with primaquine…. just lately, primaquine was included in the treatment just because we have rise of vivax malaria.*

***SSI-15, Solomon Island***

### Operational challenges and barriers to roll out current radical cure regimens

The majority of the countries had a conventional radical cure regimen in their national treatment guidelines that consisted of chloroquine for 3 days and (0.25 mg/kg) + PQ for 14 days. Few countries such as Malaysia, Cambodia and Indonesia include ACT instead of CQ while the Philippine treatment guidelines includes both drugs. Cambodia does not include a radical cure regimen in the national treatment guidelines although PQ single dose is recommended to clear *P. falciparum* gametocytes and the feasibility of implementing a (0.25 mg/kg) PQ × 14-day regimen is currently being assessed. Most countries include a 14-day radical cure regimen in their national treatment guidelines, whether the regimen is well implemented appeared questionable. Most respondents agreed that the implementation of radical cure is weak and did not have confidence in adherence.

*R: I think there are three challenges. First thing is complexity of regimen. Second one is adherence. that is quite difficult for people.*

*I: Follow-up for 14 days?*

*R: Ya, follow-up for 14 days. And the last one is need for pretesting of G6PD. Because of our supply system is not stable. That means some time we found G6PD test short of supply and Pv patients could not get radical treatment without G6PD testing.*

***SSI-10, Laos***

Reasons for poor implementation of the radical cure ranged from lack of confirmatory diagnosis of vivax malaria, fear of adverse events due to PQ, lack of wide availability of PQ, no clinical supervision strategy e.g. Directly Observed Treatment Strategy (DOTS), and DOTS itself being a barrier because either health worker or patient has to travel to receive the drug. In some countries, novel radical cure regimens were being piloted in a few selected provinces before implementing them nationwide.

*I: So, your vivax treatment regimen currently includes low dose primaquine for 14 days?*

*R: Ya, but currently only for the piloting provinces, in the early next year we will have combined ACT and primaquine for 14 days.*

***SSI-09, Cambodia***

These impediments, fear of adverse events due to PQ, limited availability of the (minimum) qualitative G6PD tests, have resulted in ‘prescription hesitancy’ among health workers.

*‘Primaquine is only available in public sector and that too only in few hospitals and high [malaria] prevalent districts only. [In] [t]The low [malaria] prevalent districts and private market, primaquine is not available. So, this availability is one of the main challenges and we know that most of the cases would go to the private sector; and those cases are treated on clinical grounds. So, as I have explained earlier that clinically diagnosed patients in the private sector won’t get any radical therapy. Second, with regards to routine clinical care to patients, their capacity, capacity of the health care providers, their reluctancy in some areas to prescribe primaquine according to national guidelines is due to absence of G6PD screening. That is again is, we try but there are medical doctors [who] won’t prescribe this primaquine without G6PD screening. Then of course compliance remains like anywhere in other countries in the world. Case compliance remains [a] challenge in Pakistan as well.’*

***SSI-16, Pakistan***

G6PD testing (at least qualitative RDTs) was a critical component in the national programs affecting the implementation of radical cure regimen. Except Malaysia and the Philippines where all new-borns undergo G6PD testing, G6PD qualitative RDT kits were not routinely available at the health centre/community levels in Laos and Vanuatu even if G6PD testing was mandatory but was perceived to restrain the use of radical cure. In other countries where G6PD test was optional, they utilized the RDTs and quantitative tests where available, and relied on clinical supervision where G6PD tests were unavailable.

*‘So, if G6PD tests are available and risk of patient [is known, G6PD test is done before providing radical cure treatment]. The program has supplied few kits in places where based on research there are high prevalence of G6PD deficiency but in most of [the] areas there are no G6PD tests. In those areas, we have mentioned in the guideline that a very thorough counselling should be done. Using annexes in our national guideline which recommends to follow up on 3, 4, 7 and 14 days of starting treatment and it has list of checklists on what to follow up, what kind of symptoms to follow up when they make contacts during those days.’*

***SSI-03, Nepal.***

There were various reasons for not including G6PD test in the routine treatment protocol of many of these countries ranging from perceptions that the prevalence of G6PD deficiency was not high in the country; no/minimal experience of haemolytic anaemia due to PQ in the past; assumed safety due to clinical supervision of patients after PQ treatment and a mental risk–benefit comparison of providing PQ with or without G6PD test. Compelling health workers to conduct G6PD testing before prescribing radical cure regimen was thought to be an important barrier to prescribing the radical cure.

*I: Why do you not ask G6PD test before initiating radical cure regimen? Is it the budget, or any other factor?*

*R: I think it’s not about budget availability. Because our government has money even to provide PCR-TB test…gene expert that is much more expensive. So, perhaps because we don’t see a lot of side effects of primaquine due to the G6PD deficient [deficiency] [in] [a] patient who takes [a] low dose of primaquine. But if we want to use the high dose of primaquine to eliminate vivax, I am sure they will start making plan and proposal and go to the central government to get it funded.*

***SSI-22, Indonesia.***

*‘….. we just give them [primaquine without G6PD test] and see how it goes.’*

***SSI-23, Myanmar.***

When the options and scenarios for G6PD testing using newer point of care quantitative tests (biosensor) were discussed, respondents were split regarding who should administer it at the community. Several respondents especially in the Western parts of Asia (India, Pakistan, Bangladesh, Nepal and Bhutan) felt that village or community health care workers should use biosensor for G6PD tests. Since these frontline health care providers prescribe and frequently administer schizontocidal treatment for the acute malaria episode, integration of the radical cure at the point of first contact holds promise to be most effective.

*‘At the moment the lab technicians [do the G6PD test], but in case there [is] be point of care testing [available] there might be possibility to test through the community health workers…[which is] also possible. … It would be good if we use the biosensor at the level of community health workers.’*

***SSI-11, Afghanistan.***

*‘We have to train Village health workers to use biosensors.’*

***SSI-22, Indonesia.***

*‘… in our situation where malaria cases are in the community level, the person who does the tests should be village health workers.’*

***SSI-21, Vietnam.***

*‘It depends on how technically [simple these tests are] and simplicity of these tests. For example, RDTs, it is being used at [the] community level by village health workers, depends on how complex it is, [if it is simple] simpler than it can be used at [the] community level.’*

***SSI-29, PNG***

In contrast, respondents from South East Asia (with the exception of Vietnam) were more reluctant to trust the primary health care workers to administer the tests and interpret the results appropriately. In addition, providing costly biosensors and test cartridges with limited shelf life (< 12 months) to village health workers who may not encounter a single vivax malaria patient for months on end appears wasteful.

*‘I don’t think it will be effective because [there is a low prevalence of vivax patients] as now patients become less and less. So, some malaria worker they won’t get any patients. …We implemented [a pilot study for radical cure with biosensor] when the health center has at least one case every month. You know to setup this system you need to train people; you need to equip health center with one fridge to keep the tests you know a lot [of] requirement. So, I don’t see it a need to start expanding to community volunteer because they don’t have a lot of cases.’*

***SSI-12, Cambodia***

*‘It’s quite hard as we have got only 576 [P. vivax] cases annually to now talk about introducing a more advanced technology approach for G6PD testing.’*

***SSI-28, Vanuatu***

### Evidence gaps for future radical cure regimens

Considering the challenges reported to be inherent in current radical cure regimen (14 days PQ) and G6PD testing, all respondents echoed a need for shorter treatment regimens. Although most respondents believed that the shorter treatment regimens with high dose PQ (7 days) and single dose Tafenoquine could resolve the adherence problem of conventional radical cure regimen, the challenges of the formative phase of evidence synthesis and the regulatory process was well recognized. Few respondents expressed confidence in the traditional radical cure regimen (0.25 mg/kg PQ × 14 days) and asked for more evidence on shorter regimens including the endorsement by WHO and national regulatory bodies.

*I: Have you heard of shorter regimens, what about high dose primaquine for 7 days?*

*R: Yes, [but] comfortable with current regimen of 14 days. Higher dose means it will be mandatory to conduct G6PD test. 14 days means we are able to follow this regimen even without G6PD testing. Even if there is haemolysis that will be of low range.*

*I: What about single dose Tafenoquine?*

*R: First thing, it has to be approved by the regulatory bodies. Number 2, there has to be G6PD test, a suitable G6PD test [needs to be] available; third thing, there again is need of operation[al] research on this implementation study just to introduce it, [it is] not possible to implement without having first time experience… without operational research.*

***SSI-06, India***

Almost all respondents stated the need for robust G6PD testing mechanisms before rolling out shorter treatment regimens. Implementation of shorter treatment regimens required the co-introduction of appropriate G6PD tests. Few respondents shared their concerns regarding the shortcomings of currently available qualitative tests and referred to the need for studies of point of care quantitative tests such as biosensors. Other respondents challenged the introduction of novel radical cure regimens and G6PD tests, pointing to programmatic hurdles in rolling out, such as constraints in human resources working in malaria, and quality and skills required to use the new quantitative diagnostic tests compared to qualitative RDTs. Also, the barriers in revising national policies were reported to be complex as the approval of novel tests involved various review processes from WHO to NMCP. Many respondents emphasized the role of Global Fund and WHO as the critical bottleneck in introducing these tests into the national programs:

*‘… honestly and very frankly they will be rolled [out] by Global Fund. I don’t [think] national or provincial malaria program are in any sort of position to introduce it. … Global Fund recommends and WHO approves it … It’s basically between WHO and Global Fund.’*

***SSI-4, Pakistan***

Nearly every respondent commented on the crucial position of the WHO in advising governments and their malaria control programmes.

*‘WHO is the only organisation whose recommendation is mostly applicable, and country will comply to their recommendations.’*

***SSI-14, Afghanistan***

*‘To be in WHO guideline you probably know how it works. So, you have to go through complete process of evidence accumulation and review and this will be based on decision of expert committee or several expert committees meeting in Geneva and then you are in WHO guideline. As simple as that.’*

***SSI-13, Cambodia***

*‘… then WHO said they don’t recommend it so we did not go forward with that trial and stuck with the 14 days … sometimes [WHO] they might give us wrong decision as well.’*

***SSI-15, Solomon Islands***

One of the essential commonalities among the respondents was the demand for evidence on feasibility of new generation POC quantitative G6PD test at the field level, for example who can/should use it (training and capacity to use it); cost implications of these machines, durability and sustainability.

*I: If you were to adopt a new biosensor for G6PD point-of-care testing, what evidence would you need to recommend it?*

*R: We need published articles, we need the data on safety and efficacy (Specificity and sensitivity), like RDTs are WHO pre-qualified, if applicable--these are the things we need first before considering it into the program. We will be asked to justify why we are recommending this new technology? Other than that, it has to pass the Health Technology Assessment and all regulatory requirements- FDA registration. We need to pilot test the technology to determine, whether our health workers can use and interpret the data correctly, its practicability, cost effectiveness, etc – before we can consider expanding it on more areas.*

***SSI-17, Philippines***

## Discussion

### Overview of findings

Although malaria continues to decline in Asia, *P. vivax* persists due to its relatively sophisticated and complex biology. The radical cure poses critical challenges for the countries embarking on vivax malaria elimination within the next decade [30]. Unlike falciparum malaria that received significant attention, vivax malaria has remained neglected for too long which has hindered the rollout of the radical cure regimen [15, 31–33]. PQ has been heterogeneously implemented across the countries in South/South East Asia and Asia Pacific [34]. Adherence issues with PQ 14 days and mandatory G6PD test in some countries was thought to impede its prescription. On the other hand, health workers did not feel that providing PQ without G6PD test was safe [35]. Shorter radical cure regimens with POC quantitative G6PD testing was often desired but with a requirement for more operational evidence.

### Policy priority towards vivax malaria

A commonality among most of the countries in Asia was how they acknowledged the lack of specific policies related to vivax malaria [34]. Policies related to community-based diagnostic tools, surveillance, pharmacovigilance, estimation of sub-microscopic reservoir, treatment guidelines, vector control strategies, adherence to radical cure regimen and the feasibility of G6PD testing were generally lacking. The neglect and under-prioritization of vivax malaria may have stemmed from the global focus on falciparum malaria [36]. The persistence of vivax malaria as opposed to the rapid decline of falciparum malaria demonstrates unequivocally the inadequacy of current malaria control strategies and thus calls for specific and additional interventions [37]. Under-prioritization affects funding and diminished funding affects the effectiveness of stakeholders. This observation is today more important than ever, as the declining malaria burden offers false hope leading to complacency by international funding bodies [38–40]. Some of the respondents are particularly concerned how competing priorities and a decline of malaria may result in malaria (including vivax malaria) losing global public health attention [40]. Increased engagement and advocacy are essential to roll out the radical cure for vivax malaria. Although delayed, the WHO responded to vivax malaria by integrating the data into World Malaria Report since 2013 [15]. The respondents considered the WHO by far the dominant stakeholder in setting policy priorities [41, 42]. The WHO and NMCPs are each partly responsible for the incomplete and lacklustre implementation of the radical cure. The sheer sloppiness of the policies addressing G6PD testing has been a major barrier for countries facing an increasing relative proportion of vivax malaria. Further evidence of the persistent neglect of vivax malaria comes from the heterogeneity of treatment regimens and G6PD tests, not to mention their implementation and availability. In many of the countries represented in this study, neither RDTs to diagnose vivax malaria, nor RDTs to diagnose G6PDd, nor PQ were available, leaving little doubt that vivax malaria has, if any, a low priority in national and international health policies.

### Operational challenges and barriers to roll out current radical cure regimen

There is consensus that robust case management of malaria, specifically the correct diagnosis and immediate adequate treatment is vital [43]. Yet the unavailability of RDTs to diagnose vivax malaria (for example in Pakistan) is a major impediment for further discourse on the optimization of radical therapy. Radical cure regimens are a quintessential minimum requirement for the adequate management of vivax malaria and not an optional add-on [44, 45].

Few countries have currently a radical cure regimen in their policy, Laos had revised the national treatment guidelines in 2016 and Cambodia is yet to introduce the radical cure in their guidelines. Even countries with the radical cure included in the national guidelines, have little or no confidence in its implementation [34, 45]. For instance, in Laos and Vanuatu, adherence to PQ × 14 days was known to be poor because G6PD test kits were not always available at the community level, even if G6PD test was mandated by the national treatment guidelines. The requirement of G6PD testing prior to prescribing PQ has discouraged health workers who often have no access to such tests. This conundrum has resulted in ‘prescription hesitancy’ in Afghanistan, Solomon Islands, Vietnam, and Pakistan. Prescription hesitancy has been echoed by a recent systematic review which found clinicians avoiding PQ in areas where the prevalence of G6PD deficiency is high [46]. Mandatory G6PD testing was heavily contested by respondents because of the constraints in health services leading to procurement bottlenecks and ultimately resulting in the under-utilization of PQ. The absence of G6PD testing leads to a familiar dilemma of having to weigh the risk of harm caused by relapsing vivax malaria versus potential haemolysis caused by 8-aminoquinolines [35]. In settings with high vivax malaria burden, high risk of relapse and poor access to health services, either decision has unintended consequences and is referred to as ‘the PQ-G6PD dilemma’ [47, 48]. Perhaps most importantly the incentives are misaligned. First, practitioners are wary that by prescribing an 8-aminoquinoline regimen they may be held responsible for a subsequent haemolytic episode. Irrespective of national customs and recommendations it seems likely that the practitioner will lose at least one client. Second, by preventing relapse they forfeit the potential income from the treatment of such disease episodes.

Some countries such as Nepal, India, Thailand, and Vietnam were more flexible, recommending G6PD testing where available and prescribing PQ without testing but with clinical supervision where not available. Providing medical advice to patients about potential adverse events and follow-up was perceived to provide an adequate safety net for prescribing PQ without G6PD test [49]. Potential adverse events following PQ administration are underreported. In most vivax endemic countries in Asia with poor health infrastructure, neither true adherence nor the adverse events related to PQ can be accurately estimated [18].

Although Laos and Vanuatu mandate G6PD testing, both suffer from inadequate supply and maintenance of test kits in the peripheral health facilities. In Laos for example, a recent study found that G6PD rapid diagnostic kits were only available at the provincial or district hospitals but not at the point of first contact with the health care system where schizontocidal therapy is prescribed [50]. To receive a course of PQ the patient would have to take the initiative to travel to the provincial or district hospital once sufficiently recovered to undertake such a trip. No recommendation for G6PD testing in Indonesia, Pakistan, and PNG was thought to be due to an inadequate health system not equipped to integrate G6PD testing. Respondents perceived PQ induced haemolysis a low risk based on past experience, and low prevalence of G6PD deficiency. Mandating G6PD tests was thought to simply impede the prescription of PQ. The perception that PQ-induced haemolysis carries a low risk for severe adverse events was reported to be one of the most prominent barriers in endorsing and adopting G6PD testing in Bangladesh and China [29].

### Evidence gaps for future radical cure regimens

The limitations of currently available regimens for the radical cure have triggered a demand for new and shorter regimens that can address these challenges [18, 19, 34]. Recent evidence suggests that a high dose, 7 day PQ regimen is highly efficacious in preventing relapse and reduces the length of treatment by half [19] but requires an accurate, POC G6PD test in place [47, 51–53]. The single dose regimen TQ was thought to be ideal but the need for accurate, quantitative G6PD testing was a prominent reservation [54]. The price for shorter treatment regimens is an accurate G6PD testing mechanism in place. Respondents thought shorter regimens may be implemented in the future, for the time being respondents were more comfortable with the conventional 14-day PQ regimen as it was familiar, in use for long time, and has WHO approval [37, 45, 46].

Shorter treatment regimens may require an accurate quantitative assessment of G6PD activity prior to administration to avoid haemolysis in heterozygous females [20, 48, 52, 55]. One promising approach to resolve this quandary is a new generation of biosensors, that are portable and can quantify the G6PD activity of a patient at the point of first contact with the health system [20, 55]. Although majority of respondents are familiar with biosensors, they requested more evidence on its performance, and more specifically, how it can be implemented in field settings. The respondents asked for more information on the operational aspects of biosensors, including cost, durability in hot humid conditions, shelf-life, portability, security, and sustainability. These findings echo recent discussions at policy forums urging for more operational evidence on the use of biosensor and practicalities associated with integrating it in routine health care [28, 56]. For instance, how the biosensors perform in the hands of field staff, whether or not field staff can routinely use it to inform the radical cure treatment algorithms, how sustainable is their integration into the routine health care and other practical issues related to its use.

Irrespective of the reservations respondents expressed regarding the performance of the WHO, the approval mechanism provided by the organization remains an essential condition for the introduction of public health interventions no matter whether new radical cure regimens or biosensors to quantify G6PD activity. Paradoxically, the very same respondents who complain about the inadequacy of vivax malaria policy recommendation, rely on WHO recommendations as a *sine qua non* for policy change. Drafting recommendations for novel radical cure regimens or G6PD tests requires a lengthy bureaucratic review process by the WHO. Nevertheless, any substantial change in the management of the radical cure for vivax malaria was thought to hinge on WHO’s recommendations and has been an established policy-change-mechanisms in most of the countries [41, 42, 56, 57]. The relevant representatives of the WHO such as the Global Malaria Programme furthermore insist that only the organization has the mandate to provide policy advice, have assigned vivax malaria historically a low priority, and is yet to build the capacity to manage vivax malaria policy recommendations. Consequently, the current absence of consistent, adequate vivax malaria policies throughout the region is ultimately a reflection of the reliance on the WHO and the inadequacy of the WHO leadership to fulfil the expectations and deliver appropriate policy recommendations in a timely fashion.

### Strengths and limitations

This study interviewed the respondents from 17 countries and thus covers the policies and challenges around implementation of radical cure regimens in most of South/South East Asia and Asia Pacific region. The wide geographical coverage carries with it a heterogeneity in the context, policies and priorities. An attempt was made to disentangle the commonalities (and differences) relevant to the countries represented in this study. Although the study explores the national perspectives on radical cure regimen, almost half of our respondents did not represent the national malaria control programmes and explicitly asked to disclaim any national/NMCP representation in this article. Although the investigators attempted to interview at least two respondents for each country in addition to reviewing national malaria reports, and WHO reports to achieve data saturation, data in this study may not always represent the national policy scenario. As policymakers and stakeholders in this study represent the heterogeneity of interest, role and influence in policy making, responses may not truly reflect the decision-making process in policy and its implementation. Respondents in this study were approached purposively using a list of participants from authors’ professional networks such as APMEN and MORU. Most of these respondents are male and may reflect the gender imbalance among policymakers and stakeholders in the region or in the list used in the study, because gender was not a criterion for selection in this study. Also, data in this study may have suffered from respondents’ interests and desirability bias. All but two interviews in this study were conducted virtually which may have missed subtle but critical nuances, facial expressions, and body language which provide ever so helpful cues in face-to-face interviews.

## Conclusion

Vivax malaria remains perhaps the last but undoubtedly the most difficult barrier to malaria elimination in South/South East Asia and Asia Pacific. Vivax malaria is complicated by its biological characteristics and the need for the radical cure regimen. The inadequate implementation of the radical cure due to the fear of adverse events and poor adherence may be overcome by POC quantitative G6PD test by enabling healthcare providers to prescribe shorter yet effective treatment regimens. Operational evidence that includes the feasibility of integrating biosensor into the administration of radical cure regimens will be critical for the roll out of the radical cure.

## Supplementary Information

**Additional file 1.** COREQ (COnsolidated criteria for REporting Qualitative research) checklist.

**Additional file 2.** Semi-Structured Interview Guide.

## Data Availability

The data is available upon request to the Mahidol Oxford Tropical Medicine Research Unit Data Access Committee (http://www.tropmedres.ac/data-sharing) complying with the data access policy (http://www.tropmedres.ac/_asset/file/data-sharing-policy-v1-0.pdf).

## Abbreviations

ACT: Artemisinin-based Combination Therapy
COREQ: COnsolidated criteria for REporting Qualitative research
COVID-19: Corona Virus Infectious Diseases-2019
CQ: Chloroquine
DFID: Department for International Development
DOTS: Directly Observed Treatment Strategy
FDA: Food and Drug Administration
G6PD: Glucose 6 phosphate dehydrogenase
G6PDd: Glucose 6 phosphate dehydrogenase deficiency
LLINs: Long-lasting insecticidal nets
MRC: Medical Research Council
NMCPs: National Malaria Control Programmes
PNG: Papua New Guinea
POC: Point of care
PQ: Primaquine
RDT: Rapid diagnostic test
SSIs: Semi-structured interviews
TQ: Tafenoquine
WHO: World Health Organization
