# Supplementary material for: Rolling out the radical cure for vivax malaria in Asia: a qualitative study among policy makers and stakeholders
Source: Malar J. 2021 Mar 23;20:164. doi: 10.1186/s12936-021-03702-5 (PMC7987122; doi:10.1186/s12936-021-03702-5)
Supplement: Supplementary file 2 — Additional file 2. Semi-Structured Interview Guide. [file 12936_2021_3702_MOESM2_ESM.pdf]

Appendix I: Semi-Structured Interview guide

Study title: Factors affecting implementation of radical cure therapy for vivax malaria in South East Asia and Asia Pacific—a qualitative study among policy makers and stakeholders

Semi-structured interviews

(These are only guides to prompt interviewer and interviewee to adhere to the topic. Please feel free to add the themes and concerns as you see fit)

AUDIO FILE NAME:

Introduction (read out):

I am ………………… from Mahidol-Oxford Tropical Medicine Research Unit, Bangkok. I am a researcher and I am approaching you to discuss on radical cure therapy for vivax malaria in Myanmar. As you know the countries in the South East Asia and Asia Pacific are heading towards malaria elimination, it is important to know the current epidemiological burden of malaria, policy priority, and bottlenecks for malaria elimination. Since the proportion of vivax malaria in the region is high compared to the falciparum malaria, it poses unique challenges and opportunities. In this study, I am interested to explore your perspectives on vivax malaria, current policies guiding treatment plans and the strategy to achieve malaria elimination in the nearest future. Your opinion and perspectives will guide us to specifically design the future research to generate evidence that can ultimately help policymakers to revise/enforce the existing policy or the implementation plan to cure the vivax malaria. There are no direct benefits attached to your participation in this study. I will respect your time and if you feel uncomfortable or distressed due to questions asked or the time consumed, you can inform me at any time and I will immediately stop the interview. Also, you can drop out of the interview at any time and you do not have to give me reasons for it. If you consent to participate, I would like to inform you that this interview will be anonymized, the information you will provide will be confidential. Please feel free to ask questions related to this study at any time before making a decision. If you agree, I would like to audio-record this interview for transcription and analysis.

SECTION-I: General Information

<table>
<tr><td>1. Age</td><td colspan="5"></td></tr>
<tr><td>2. Gender</td><td colspan="5"></td></tr>
<tr><td>3. Country</td><td colspan="5"></td></tr>
<tr><td>4. Qualification</td><td colspan="5"></td></tr>
<tr><td>5. Type of job (government/private)</td><td colspan="5"></td></tr>
<tr><td rowspan="2">6. Workplace (tick mark)</td><td>Central</td><td>Province</td><td>District</td><td>Community</td><td>Other</td></tr>
<tr><td></td><td></td><td></td><td></td><td></td></tr>
</table>

	7. Characteristics of your work (e.g. policy planning/ supervision/ Administration/clinical care/research	
**SECTION-II: Strategies and policies related to malaria elimination**		
	**Guide**	
	8. When is your country eliminating malaria? (which year is malaria elimination planned to be completed?)	
	9. Could you describe the malaria burden in your country? /region?	
	10. How much of the malaria burden is due to vivax in your country? /region?	
	11. Can you explain the malaria elimination strategy in your country? /region?	
	12. What are the current bottlenecks that you think are important to consider to achieve malaria elimination?	
**SECTION-III: Policies and treatment practice related to vivax malaria**		
	13. Are there specific policies related to vivax malaria in your country? /region?	
	14. What are the current treatment plans/treatment regimens for vivax malaria in your country? /region?<br>*Note: If the treatment regimen does not have radical cure therapy, ask why? If Yes, proceed with the questions below: DHA piperaquine +14days PQ*	
	15. Does your treatment regimen for vivax malaria include radical therapy with an 8 aminoquinoline such as Primaquine?	

	16. Does your treatment regimen for vivax malaria mandate a routine G6PD test before initiating radical treatment?
	17. If YES: What do you currently use for routine G6PD testing? (Prompt: FST, RDTs such as Care Start, BinaxNOW, …../Spectrophotometry/Genetic testing)
	18. If YES: In your opinion, do you think the health workers follow radical treatment regimen for vivax malaria with G6PD testing?
	19. What do you think are the challenges in adopting radical therapy with G6PD testing? (prompt: vivax malaria as a priority, perceived severity due to vivax malaria, complexity of the regimen, adherence, need for a pre-testing of G6PD, health human resources capable of supervising the treatment……)
	20. Would the newer point-of-care testing and shorter treatment regimen resolve the challenges currently you have?
**SECTION IV: Options and scenario for G6PD testing**	
	21. For a routine vivax treatment, who does G6PD test at the community? (Prompt: no one, village health/malaria workers, health/physician assistant, doctor, lab personnel)
	22. Are you familiar with the novel point-of-care quantitative G6PD testing such as biosensor?
	23. If you were to adopt a new biosensor for G6PD point-of-care testing, what evidence would you need to recommend it?
	24. If such biosensors are available who could/should do the test? (prompt: village health workers, health centre staff, nurses, lab personnel, doctor)
	25. What evidence would you like to see to allow village health workers to use biosensor? (Prompt: cost of biosensor, who can do the test? Who is going to pay for the training?)

SECTION V: Options and scenario for radical cure	
	26. Have you come across the recent evidence on shorter treatment regimen and single dose radical treatment for cure for vivax malaria?
	27. One option is a use of a single dose Tafenoquine for radical cure. If you were to adopt this in the national treatment protocol, what evidence would you like to see?
	28. What challenges do you see in adopting a single dose Tafenoquine?
	29. There is recent evidence on the efficacy of a shorter regimen of Primaquine such as 7-days high dose regimen compared to the standard 14-days regimen. Can the 7-day Primaquine regimen be implemented in your country /region?
	30. What additional evidence could convince National Malaria Control Programs to adopt this regimen?
	31. Would a 14-day Primaquine regimen be more convincing than a 7-day regimen or tafenoquine in your country? Why?
Can you provide us your free opinion on current gaps and needed evidence on radical cure therapy for vivax malaria?	
The End	
